# Correlation Between Salivary Microbiome of Parotid Glands and Clinical Features in Primary Sjögren’s Syndrome and Non-Sjögren’s Sicca Subjects

**DOI:** 10.3389/fimmu.2022.874285

**Published:** 2022-05-04

**Authors:** Donghyun Kim, Ye Jin Jeong, Yerin Lee, Jihoon Choi, Young Min Park, Oh Chan Kwon, Yong Woo Ji, Sung Jun Ahn, Hyung Keun Lee, Min-Chan Park, Jae-Yol Lim

**Affiliations:** ^1^ Department of Otorhinolaryngology, Yonsei University College of Medicine, Seoul, South Korea; ^2^ Yonsei University College of Medicine, Seoul, South Korea; ^3^ Gangnam Severance Hospital, Yonsei University College of Medicine, Seoul, South Korea; ^4^ Division of Rheumatology, Department of Internal Medicine, Yonsei University College of Medicine, Seoul, South Korea; ^5^ Department of Ophthalmology, Institute of Vision Research, Yonsei University College of Medicine, Seoul, South Korea; ^6^ Department of Radiology, Yonsei University College of Medicine, Seoul, South Korea

**Keywords:** Sjögren’s syndrome, microbiome, microbial diversity, saliva, parotid glands, bioinformatics

## Abstract

Recent studies have demonstrated that the oral microbiome in patients with Sjögren’s syndrome (SS) is significantly different from that in healthy individuals. However, the potential role of the oral microbiome in SS pathogenesis has not been determined. In this study, stimulated intraductal saliva samples were collected from the parotid glands (PGs) of 23 SS and nine non-SS subjects through PG lavage and subjected to 16S ribosomal RNA amplicon sequencing. The correlation between the oral microbiome and clinical features, such as biological markers, clinical manifestations, and functional and radiological characteristics was investigated. The salivary microbial composition was examined using bioinformatic analysis to identify potential diagnostic biomarkers for SS. Oral microbial composition was significantly different between the anti-SSA-positive and SSA-negative groups. The microbial diversity in SS subjects was lower than that in non-SS sicca subjects. Furthermore, SS subjects with sialectasis exhibited decreased microbial diversity and Firmicutes abundance. The abundance of Bacteroidetes was positively correlated with the salivary flow rate. Bioinformatics analysis revealed several potential microbial biomarkers for SS at the genus level, such as decreased *Lactobacillus* abundance or increased *Streptococcus* abundance. These results suggest that microbiota composition is correlated with the clinical features of SS, especially the ductal structures and salivary flow, and that the oral microbiome is a potential diagnostic biomarker for SS.

## Introduction

Sjögren’s syndrome (SS), a chronic autoimmune disease, mainly affects the exocrine glands, especially the salivary and lacrimal glands (1). Lymphocytic infiltration of exocrine glands leads to obstructive sialadenitis and glandular hypofunction (2, 3). The diagnostic tests for SS include unstimulated salivary secretion test and Schirmer test to assess glandular hypofunction, serological tests to detect antibodies (such as anti-Ro/SSA and anti-La/SSB antibodies), and labial salivary gland biopsy (LSGB) for evaluating autoimmunity. Although the pathophysiology of SS is unclear, genetic, environmental, and hormonal factors can induce SS. Currently, the crosstalk between innate immune cells, adaptive immune cells, and non-immune cells (such as glandular epithelial cells) is hypothesized to be involved in the pathophysiology of SS (4, 5).

The microbiome, which represents a group of microorganisms in the mammalian host, regulates various host physiological processes, including the immune response. Several studies have reported the importance of the microbiome and its interaction with the host immune system in maintaining tissue homeostasis and mediating the pathophysiology of gastrointestinal tract, oral cavity, female reproductive organ, and skin diseases and systemic disorders (6, 7). Dysbiosis is characterized by the loss of microbial diversity. The disruption of the equilibrium between pathogenic and commensal microorganisms can increase the susceptibility of the host to systemic inflammatory diseases. Various factors, such as use of antibiotics, diet and oral hygiene, and salivary gland dysfunction can contribute to dysbiosis, which leads to a systemic proinflammatory condition and the development of autoimmune diseases.

The oral and gut microbial profiles are altered in patients with SS (8–11). Most studies have focused on the gut microbiome composition, which is determined using stool samples, and reported the indirect role of the gut microbiome in the severity of remote salivary gland diseases. Some studies have examined the oral microbiome (12) using saliva samples obtained through mouth washing or spitting, which is an easy and non-invasive sample collection method. However, saliva composition can be affected by environmental factors, and researchers should minimize the influence of diet, smoking, medication, and hygiene (11, 13). Furthermore, the saliva samples obtained from washout or spitting may comprise heterogeneous microbiota from the tongue, tooth, gingiva, tonsils, and buccal mucosa. Thus, there is a need to develop a standardized saliva collection method to obtain gland-specific saliva (14), which will enable the elucidation of the correlation between the microbiome and pathophysiology of SS.

In this study, saliva was collected directly from the parotid gland (PG) through ductal probing and lavage. The microbial community in saliva was investigated with the amplicon sequence variant (ASV) method, which infers exact sequence variants at high resolution and provides a more detailed landscape of diversity (15). To our knowledge, this study is the first to investigate the microbiome in PG saliva in an effort to mitigate bias caused by environmental variation and to reduce the chances of oral flora contamination, which has not been employed in previous microbiome studies. Furthermore, patients from the homogenous sicca cohort were prospectively enrolled. The microbiome composition of SS sicca and non-SS sicca subjects was comparatively analyzed. Additionally, the correlation between microbiome composition and clinical manifestations was examined using statistical and machine-learning-based methods. The results showed that the salivary microbial diversity was significantly dysregulated in patients with SS. Several disease-specific microbial features were also identified. The microbiome composition was significantly correlated with anti-SSA/Ro positivity and the radiological findings of the SS sicca group. These findings suggest that the microbiome has a potential role in the pathogenesis of SS and the microbiome composition may indicate the biological status in SS subjects.

## Materials and Methods

### Sicca Patient Cohort

The Institutional Review Board of the Gangnam Severance Hospital (IRB No. 3-2020-0160) approved this study. The sicca cohort comprised patients who visited the clinics of ENT, Eye, or Rheumatology in the Gangnam Severance Hospital with sicca symptoms, including xerostomia and xerophthalmia. The patients were administered a questionnaire assessing the following: “I have trouble swallowing solid food,” “I wake up while sleeping for a cup of water,” “I feel dryness in my mouth while eating,” “I need a sip of water to swallow solid food,” “I usually drink water to reduce dryness,” “I have trouble eating dry food,” “I feel dryness in my mouth while speaking,” and “I feel dryness in my mouth while chewing” (16). The patients with definite subjective dryness in their mouth or eyes were then enrolled in our sicca patient cohort. The saliva samples were collected for microbiome analysis from 43 patients. Of these 43 samples, 32 quality-controlled samples in which the concentration of the complementary DNA (cDNA) library was > 5 ng/μL, were used for the final analysis. Among the 32 samples, 23 were from patients with primary SS sicca who were diagnosed according to the recent ACR/EULAR classification criteria revised in 2016 (17), while nine were from non-SS sicca subjects who did not fulfill the diagnostic criteria. Patients with SS sicca exhibited positive anti-Ro/SSA results or a focus score in the LSGB sample of more than 1 foci/4 mm^2^ with at least one of the aberrant items related to salivary or lacrimal glandular hypofunctions. Pathologists and radiologists who were blinded to the clinical and laboratory findings in this study analyzed the focus score in the LSGB samples and magnetic resonance (MR) sialography features of PGs. The exclusion criteria were as follows: history of irradiation of the head and neck area; radioactive iodine treatment for thyroid cancers; salivary gland surgery; other autoimmune diseases (potential secondary SS). Although some patients did not complete the radiological or laboratory examinations due to the coronavirus disease-2019 (COVID) pandemic, their clinical data were included for analysis once the statistical comparison was allowed.

### Saliva Sampling

The saliva samples were obtained from both PGs through salivary gland lavage. Patients were instructed to refrain from eating, drinking, and smoking for 2 h before salivary gland lavage. To reduce the chances of potential contamination, sample collection methods were performed as follows: The subjects rinsed their mouth with bottled water before sample collection. Citric acid (sialogogue) was administered to the floor of the mouth to stimulate salivation. After a small amount of saliva was secreted by PG massage, a rubber angiocatheter (Becton Dickinson Medicals Ltd, Singapore) connected to a syringe filled with 1 mL of physiological saline was gently inserted into the orifice of the PG at the opposite side of the upper second molar tooth. Gentle lavage was performed and saliva was regurgitated through the negative pressure of the syringe with PG massage. At least 0.1 mL saliva sample was collected from each side of the PG. Two syringes containing saliva samples from both sides were transported to the laboratory on dry ice. The saliva samples were aliquoted evenly into 1.5 mL Eppendorf tubes (Eppendorf, Hamburg, Germany) and stored at −80°C.

### MR Sialography

**Figure 1:**
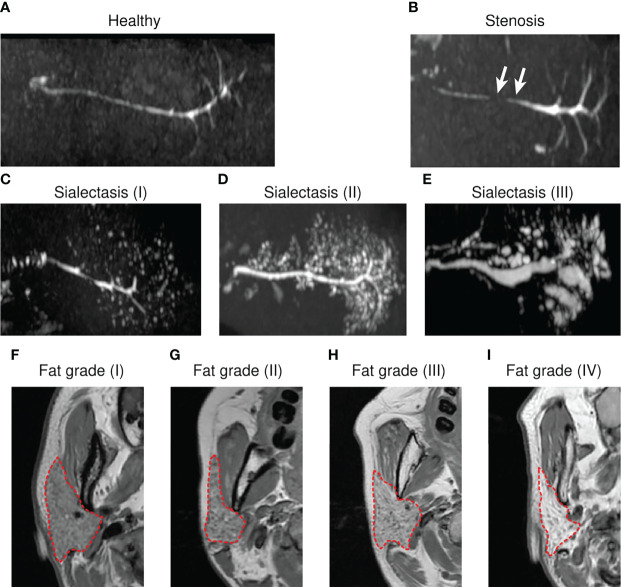
Representative magnetic resonance (MR) sialography images and grading system for fat deposition in the parotid glands. MR sialography images of healthy **(A)**, stenosis **(B)**, or sialectasis **(C–E)** ductal types. White arrows indicate narrowing of the salivary gland duct. For sialectasis, grades are represented in parentheses. **(F–I)** Characteristic appearances of fat signals on T1 weighted images (T1WIs). **(F)** Grade 1, normal parotid gland or sparse distribution of streaks-like fat signals. **(G)** Grade 2, diffusive distributed, honeycomb-like fat signals. **(H)** Grade 3, less than 50% of the total area of the whole parotid gland. **(I)** Grade 4, massively homogeneously distributed fat signal. Red dashed oulines indicate fat regions of T1WIs and were considered in fat grading.

MR examinations were performed using a 3.0T MRI unit (Discovery 750; GE Healthcare, Milwaukee, WI) equipped with a quadrature head coil. The magnetic resonance imaging protocol was as follows. Axial T2 weighted IDEAL water imaging was performed using the following parameters: repetition time (TR), 7600 ms; effective echo time (TE), 85 ms; matrix, 320 × 256; field of view, 20 cm; section thickness, 3 mm; flip angle, 111; asymmetric echo shifts, −π/6, π/2, 7π/6. Axial T1 weighted images (T1WI) were obtained with the following parameters: TR, 780 ms; TE, minimal; matrix, 320 × 256; field of view, 20 cm; section thickness, 3 mm. MR sialography was performed using a three-dimensional fast-recovery fast spin-echo sequence in the axial plane (TR, 2000 ms; TE, 695.5 ms; echo train length, 110; field of view, 20 cm; matrix, 320 × 320, thickness, 1 mm). Salivation was stimulated by intraorally applying a sialogogue to enhance the visualization of the ductal structures. Sialography images were generated by creating a maximum-intensity projection. The data volume was reduced by eliminating the post peripheral sections with a high signal from the skin adjacent to the coil and removing the volume that included the eye and cerebrospinal fluid. Based on the MR sialography, structural deformation was assessed (**Figure 1**). Sialectasis was defined based on the following Tonami’s criteria (18): Stage 1, punctuate with a diameter of ≤ 1 mm; Stage 2, globular with a diameter of 1–2 mm; Stage 3, cavity with a diameter of > 2 mm. Fat stage was evaluated based on the characteristic appearance of fat signals on T1WI and T2W IDEAL water imaging and using the grading methods reported by Izumi and Regier (19, 20). An iterative least-squares decomposition algorithm was employed to output a fat fraction map and an R2* map (21). A circular ROI was drawn within the PG. The ROIs were placed in the same position on the fat fraction map and R2* map.

### Salivary Gland Function Tests

Salivary flow tests and salivary gland scintigraphy were performed to determine the salivary gland function. A salivary flow test was conducted for 5 min by a nurse practitioner. The weight of the paper cup before and after the saliva was spit into it was measured. All patients were instructed not to eat, drink, or smoke for 2 h before saliva collection. For stimulated saliva collection, patients were administered 500 mg of ascorbic acid (sialogogue) for 4 min and instructed to continue spitting saliva for another 5 min into a second pre-weighed paper cup. The weight of the saliva was divided by 5 min to measure the salivary flow rates (SFRs) under unstimulated (UWMSFR, unstimulated whole mouth salivary flow rate) and stimulated (SWMSFR, stimulated whole mouth salivary flow rate) conditions.

Salivary gland scintigraphy was conducted using pertechnetate and Siemens gamma camera equipment as reported previously (22). The images were obtained for 20 min immediately after pertechnetate IV injection. Additionally, 5-min images were captured after stimulation with an oral sialogogue. At 1 min post-injection, the background count was checked until it reached the maximum count. The background count was the minimum when salivary secretion was stimulated after 20 min. The uptake ratio (UR) was calculated as the ratio of the maximum count to the background count. Tmin was defined as the time interval between the maximum and minimum counts. The maximum accumulation (MA) and maximum secretion (MS) were calculated as follows: MA = (maximum count − background count)/maximum count × 100 (%); MS = (maximum count − minimum count)/maximum count × 100 (%).

### Analysis of Salivary Microbiome Using 16S rRNA Sequencing

Saliva samples were stored at −80°C before sequencing. DNA was extracted from saliva samples using the DNeasy PowerSoil kit (Qiagen, Hilden, Germany), following the manufacturer’s instructions. The extracted DNA was quantified using Quant-IT PicoGreen (Invitrogen, Waltham, MA) and used for library construction according to the Illumina 16S metagenomic sequencing library protocols. To amplify the V3 and V4 regions of 16S rRNA, the following primers with Illumina adapter overhang sequences were used: V3-F: 5′-TCG TCG GCA GCG TCA GAT GTG TAT AAG AGA CAG CCT ACG GGN GGC WGC AG-3’, V4-R: 5’-GTC TCG TGG GCT CGG AGA TGT GTA TAA GAG ACA GGA CTA CHV GGG TAT CTA ATC C-3’. The purified PCR products were quantified using quantitative real-time polymerase chain reaction (qRT-PCR) according to the qPCR Quantification Protocol Guide (KAPA Library Quantification kits for Illumina Sequencing platforms). The amplicons were subjected to quality control using the TapeStation D1000 ScreenTape (Agilent Technologies, Waldbronn, Germany). Paired-end (300 bp each) sequencing was performed using the MiSeq™ platform (Illumina, San Diego, CA). All the above procedures were performed by Macrogen Inc. (Seoul, Republic of Korea).

### Bacterial 16S rRNA Sequence Analysis and Taxonomy Assignment

ASVs in the paired-end sequence files were identified using the DADA2 pipeline (v1.21) (23). Briefly, sequence reads were filtered with expected error thresholds of 2 (start) and 5 (end) of each read. Filtered reads were de-replicated and denoised using the default settings in DADA2. After merging the paired reads and removing the chimeras, taxonomy was assigned using the Silva reference database (v138.1) (24). The Ape package (v5.5) was used to construct the phylogenetic tree. All the above analyses were performed in the R environment (v4.1).

### Alpha Diversity and Beta Diversity

Phyloseq objects were prepared with phyloseq (v1.37) and used for diversity analysis. Taxa that were only observed once in the entire dataset or appeared in one sample were excluded from further analysis. The observed ASVs, Shannon index, and Simpson’s diversity index were used to assess alpha diversity. Statistical analysis and data visualization were performed using Prism 7 software (GraphPad, San Diego, CA, USA). To compare the two groups, parametric t-test with Welch’s correction or nonparametric Mann-Whitney test was performed based on Gaussian distribution. Differences were considered significant at p < 0.05. Parametric one-way analysis of variance (ANOVA) or nonparametric Kruskal-Wallis test was performed based on Gaussian distribution for comparing more than three groups. The false discovery rate was calculated using the Benjamini-Hochberg procedure. An adjusted p-value of less than 0.05 was considered significant. Normality tests were performed using the D’Agostino-Pearson omnibus normality test or the Shapiro-Wilk normality test. The Bray-Curtis distance method was applied to assess beta diversity, and the results were plotted in ordination with principal coordinate analysis. A permutational ANOVA method was used to test the significance between groups based on the distance matrix. All statistical analyses were performed using the ordinate function in the phyloseq package or the MicrobiomeAnalyst tool (https://www.microbiomeanalyst.ca) (25).

### Differential Microbiome Analysis

Differentially abundant ASVs between non-SS and SS subjects were identified using the following two methods: DESeq2 (26) and metagenomeSeq (27). DESeq2 (v.1.33) and metagenomeSeq (v.1.35) were performed in the R environment. Results from different methods were collected, and intersections at the genus level were plotted in a Venn diagram using InteractiVenn (28) (http://www.interactivenn.net). Logistic regression was performed using the run_sl function in the MicrobiomeMarker package (29). Briefly, the abundance of each genus was transformed to log10(1+x), and the relative abundance was normalized with total sum scaling. The top eight genera, which had a feature importance score of more than 10, were used to perform logistic regression. Logistic regression performed with more than eight genera did not significantly improve analysis outcomes. The receiving operating characteristic curve was plotted with the plot_sl_roc function in the MicrobiomeMarker package.

### Data Availability

The 16S rRNA sequencing data were deposited in the Sequence Read Archive of the National Center for Biotechnology Information with the BioProject accession number of PRJNA804331.

## Results

### Patient Demographics and Clinical Features Between the SS and Non-SS Sicca Groups

All study patients were female with a mean age of 51.4 years. The demographic and clinical characteristics of the study patients are shown in **Tables 1** and **2**. The SS sicca and non-SS sicca groups comprised 23 and nine patients, respectively (**Table 1**). Among the SS subjects, 15 (65.2%) tested positive for anti-SSA/Ro antibodies and 23 (100%) exhibited lymphocyte infiltration in the LSGB samples. Of the 23 SS subjects, 18 (78.3%) and 13 (56.5%) exhibited dry mouth and eyes, respectively. All patients in the non-SS sicca group exhibited dry mouth, while 5 (55.6%) exhibited dry eyes. All patients in the SS cohort group were confirmed to show more than a score of 4 according to the weight of the new criteria established by the ACR and EULAR in 2016, whereas all non-SS cohort patients showed negative findings in both anti-SSA/Ro antibodies and lymphocyte infiltration in the LSGB samples, with a total score less than 4.

**Table 1:** Patient characteristics of the study cohort.

*Demographic*	*Non-SS (n = 9)*	*SS (n = 23)*
***Age*,** *mean ± SD*	50.33 ± 15.06	51.83 ± 13.41
***Dry mouth*** *, n (%)*		
* No*	0 (0.00)	5 (21.74)
* Yes*	9 (100.00)	18 (78.26)
***Dry eye*** *, n (%)*		
* No*	4 (44.44)	10 (43.48)
* Yes*	5 (55.56)	13 (56.52)
***Parotid swelling/pain*** *, n (%)*		
* No*	2 (22.22)	9 (39.13)
* Yes*	7 (77.78)	14 (60.87)
***Focus score*** *, n (%)*		
* 0*	9 (100.00)	0 (0.00)
* 1*	0 (0.00)	10 (43.48)
* 2*	0 (0.00)	7 (30.43)
* 3*	0 (0.00)	6 (26.09)
***Anti-SSA*** *, n (%)*		
* Negative*	9 (100.00)	8 (34.78)
* Positive*	0 (0.00)	15 (65.22)
***Anti-SSB*** *, n (%)*		
* Negative*	9 (100.00)	18 (78.26)
* Positive*	0 (0.00)	5 (21.74)
***Xerogenic medication*** *, n (%)*	6 (66.67)	4 (17.39)
***PPI*** *, n (%)*	6 (66.67)	1 (4.35)
***DMARD*** *, n (%)*	0 (0.00)	1 (4.35)
***NSAID*** *, n (%)*	4 (44.44)	3 (13.04)
***MTX*** *, n (%)*	1 (11.11)	0 (0.00)
***Anti-malarial agent*** *, n (%)*	1 (11.11)	12 (57.14)
***Corticosteroids*** *, n (%)*	4 (44.44)	4 (17.39)
***Smoking*** *, n (%)*		
* Yes*	0 (0.00)	1 (4.35)
* No*	8 (88.89)	12 (57.14)
* Unknown*	1 (11.11)	10 (43.48)
***Infection history*** *, n (%)*	0 (0.00)	2 (8.70)
***Dental loss*** *, n (%)*	0 (0.00)	1 (4.35)
***Antibacterial mouth wash*** *, n (%)*	3 (33.33)	4 (17.39)

SS, Sjögren’s syndrome; SD, standard deviation; PPI, proton pump inhibitor; DMARD, disease-modifying anti-rheumatic drug; NSAID, non-steroidal anti-inflammatory drug; MTX, methotrexate.

**Table 2:** Clinical features of non-SS and SS cohorts.

Salivary gland assessments			Non-SS	SS	p-value
Salivary flow rate (mL/min)			(*n* = 3)	(*n* = 5)	
	**UWMSFR**, mean ± SD		1.03 ± 0.79	0.28 ± 0.29	0.07
	**SWMSFR**, mean ± SD		1.93 ± 0.76	0.74 ± 0.47	0.1
**Salivary scintigraphy**			**(*n* = 8)**	**(*n* = 19)**	
	**Uptake ratio**, mean ± SD		4.46 ± 1.46	4.68 ± 5.16	0.22
	**Maximum accumulation** (%), mean ± SD		55.15 ± 23.62	45.66 ± 17.98	0.08
	**Maximum stimulation** (%), mean ± SD		53.89 ± 22.84	36.14 ± 23.36	0.02*
	**Time to minimum count** (min), mean ± SD		4.38 ± 1.41	4.32 ± 1.10	0.47
**MR sialography**			**(*n* = 6)**	**(*n* = 19)**	
	**PG ductal type**, *n* (%)				0.02*
	0 (healthy)		2 (33.33)	3 (15.79)	
	1 (stenosis)		4 (66.67)	4 (21.05)	
	2 (sialectasis)		0 (0.00)	12 (63.16)	
				
	**PG sialectasis grade**, mean ± SD		n/d	1.89 ± 0.82	
				
	**PG fat stage**, mean ± SD		2.25 ± 1.48	2.53 ± 0.96	0.62
	**PG fatQ_R**, mean ± SD		35.95 ± 16.71	39.61 ± 18.43	0.66
	**PG fatQ_L**, mean ± SD		36.12 ± 17.06	41.28 ± 20.33	0.55
	**SMG fat stage**, mean ± SD		1 ± 0	2.47 ± 1.22	0.009**

*p < 0.05; **p < 0.01; SS, Sjögren's syndrome; SD, standard deviation; MR, magnetic resonance; PG, parotid gland; SMG, submandibular gland; fatQ, fat fraction; n/d, not determined.

UWMSFR in the SS (0.28 ± 0.29 mL/min) group was not significantly different from that in the non-SS group (1.03 ± 0.79 mL/min) (**Table 2**). Salivary gland scintigraphy parameters, such as UR, MA, and Tmin were not significantly different between the two groups. However, MS was significantly reduced in the SS cohort. Next, the MR sialography features of PGs were analyzed (**Figures 1A**–**E** and **Table 2**) and radiological structural deformities were compared between the two groups. In the PGs of SS patients, the sialectactic dilated duct was prominent in 12 (63.16%) glands. However, none of the non-SS subjects exhibited PG sialectasis. The fat deposition in the submandibular gland (SMG) was increased in the SS cohort, whereas PG fat depositions were not significantly different between the two groups (**Figures 1F**–**I** and **Table 2**).

### Anti-SSA-Positive SS Sicca Subjects Exhibited Decreased Microbial Diversity

**Figure 2:**
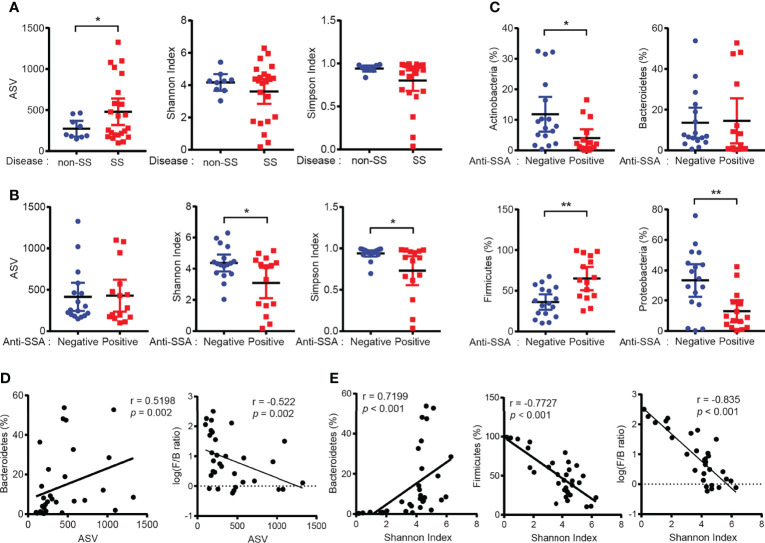
Dysbiosis of salivary gland microbiome in patients with Sjögren’s syndrome (SS). **(A)** ASV (left), Shannon index (center), and Simpson index (right) of PG saliva microbiome in non-SS (*n* = 9) and SS (*n* = 23) subjects. **(B)** ASV (left), Shannon index (center), and Simpson index (right) of PG saliva microbiome in the anti-SSA-negative (*n* = 17) and anti-SSA-positive (*n* = 15) groups. **(C)** The abundance of the top four phyla in the PG saliva microbiome was compared between the anti-SSA-negative and anti-SSA-positive groups. **(D)** Correlation between ASV and abundance of Bacteroidetes (left) and log-scaled Firmicutes/Bacteroidetes ratio (F/B ratio, right) (*n* = 32). **(E)** Correlation between Shannon index and proportion of Bacteroidetes (left) or Firmicutes (middle) and log-scaled F/B ratio (right) (*n* = 32). Data are represented as mean ± 95% confidence interval. *p < 0.05, **p < 0.01.

To investigate the correlation between the microbiome and the biological features of SS, PG saliva collected through PG lavage was subjected to bacterial 16S rRNA sequencing. The observed ASV, which was inferred using DADA2, in the SS group was higher than that in the non-SS group. Alpha diversity indices, including Shannon and Simpson indices, were not significantly different between the two groups (**Figure 2A**). Meanwhile, alpha diversity was significantly downregulated in the anti-SSA-positive group. This indicated that the microbiota in PG saliva exhibited low diversity and that it was correlated with anti-SSA-positivity (**Figure 2B**). The observed ASV and alpha diversity were not significantly different when the groups were compared based on the focus score (**Supplementary Figure 1A**).

Next, the microbial abundance at the phylum level was examined in patients with SS. The abundance of the phylum Firmicutes in the anti-SSA-positive group was higher than that in the anti-SSA-negative group. In contrast, the abundance of the phyla Actinobacteria and Proteobacteria in the anti-SSA-positive group was significantly lower than that in the anti-SSA-negative group (**Figure 2C**). In addition, the abundance of the phylum Firmicutes in patients with SS was also higher than that in non-SS subjects (**Supplementary Figure 1B**). The observed ASV was significantly correlated with the abundance of the phylum Bacteroidetes (**Figure 2D**). Meanwhile, the abundance of the phylum Firmicutes was negatively correlated with the Shannon index, whereas that of the phyla Bacteroidetes and Actinobacteria was positively correlated (**Figure 2E** and **Supplementary Figure 1C**). Additionally, ASV and microbial diversity were negatively correlated with log-scaled Firmicutes/Bacteroidetes ratio (F/B ratio, **Figures 2D, E**). Rarefaction curves further indicated that our sequencing depth was sufficient to reach maximum coverage (**Supplementary Figure 1D**). Decreased diversity and alteration of phylum abundance in anti-SSA-positive group were also observed when only SS samples were used for analysis (**Supplementary Figures 2A**–**D**). Altogether, these findings suggest that the PG saliva microbiome exhibits low diversity dysbiosis in patients with SS and that this is correlated with biological markers of SS (anti-SSA antibodies).

### Correlation of the Salivary Microbiome With Clinical, Functional, and Radiological Features

**Figure 3:**
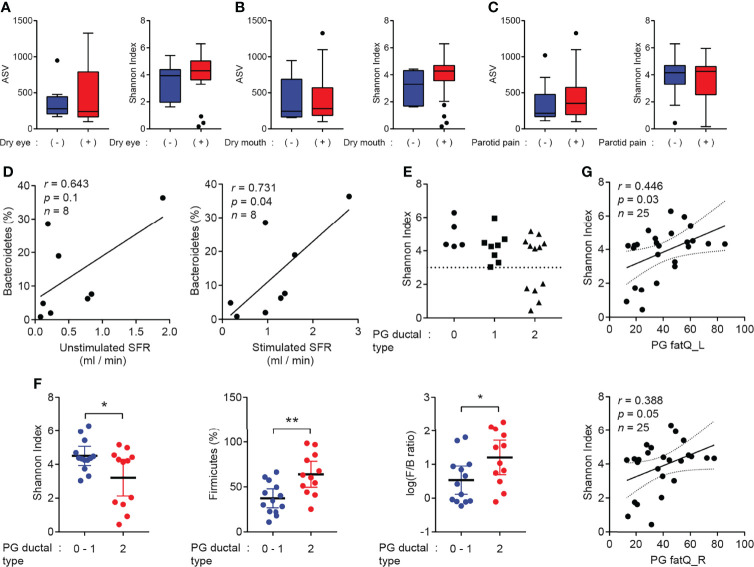
Correlation of salivary gland microbiome with clinical indicators. **(A–C)** ASV (left) and Shannon index (right) were compared based on the presence of dry eye symptoms (negative, *n* = 14; positive, *n* = 18) **(A)**, dry mouth symptoms (negative, *n* = 5; positive, *n* = 27) **(B)**, or parotid swelling/pain/discomfort (negative, *n* = 11; positive, *n* = 21) **(C)**. **(D)** Correlation between unstimulated whole mouth salivary flow rate (UWMSFR, left), stimulated whole mouth salivary flow rate (SWMSFR, right), and abundance of Bacteroidetes (*n* = 8). Solid lines indicated linear regression of data. **(E)** Ductal type of parotid gland (PG) was graded using magnetic resonance sialography images. Shannon index was compared among different groups of PG ductal type (0 = normal, 1 = stenosis, and 2 = sialectasis). Dotted line represents a Shannon index of 3. **(F)** Shannon index (left), abundance of Firmicutes (middle), and log-scaled F/B ratio (right) were compared between PG ductal types 0–1 and PG ductal type 2. **(G)** Correlation between fat deposition in left parotid gland (top), fat deposition in right PG (bottom), and Shannon index (*n* = 25). Solid lines indicate linear regression of data, while dotted lines indicate confidence intervals of 95%. Data are represented as mean ± 95% confidence interval. *p < 0.05, **p < 0.01.

To evaluate the clinical implications of the PG saliva microbiome in patients with SS, the correlation of the microbiota composition with clinical (symptoms), functional (SFR), and radiological (MR sialography) features was examined. The ASV and alpha diversity were not significantly different between the groups based on clinical symptoms, including dry eye, dry mouth, and parotid swelling or pain (**Figures 3A**–**C**). Among patients who were tested, SWMSFR was positively correlated with the abundance of the phylum Bacteroidetes (**Figure 3D**). Interestingly, we found that a significant decrease in the Shannon index (less than 3) was only observed in patients with sialectactic duct (**Figure 3E**). The Shannon index, the abundance of Firmicutes, and F/B ratio were significantly different when the groups were compared based on PG ductal types 0 (healthy) – 1 (stenosis) versus 2 (sialectasis) (**Figure 3F**). Additionally, fat deposition in the left or right PG was positively correlated with the Shannon index (**Figure 3G**). Other functional and radiological features, such as salivary gland scintigraphy parameters (US, MA, MS, and Tmin), PG sialectasis grade, PG fat stage, and SMG fat stage were not correlated with the microbiota composition in patients with SS (**Supplementary Figures 3A, B**, and data not shown). These findings indicate that distinct clinical features, including PG ductal deformity (sialectasis) and consequent salivary stasis, may be correlated with PG microbial dysbiosis and enrichment of Firmicutes in patients with SS. Additionally, high SFR without ductal dilation and fatty degeneration may be correlated with the relative abundance of Bacteroidetes and microbial diversity, respectively.

### Downregulation of *Lactobacillus* Is a Characteristic Feature in Patients With SS

**Figure 4:**
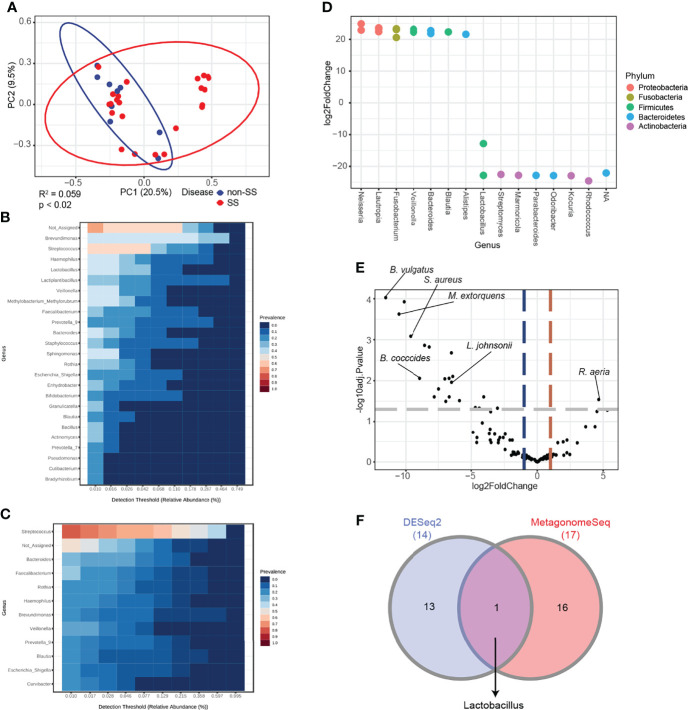
Differential analysis revealed that *Lactobacillus* is a potential biomarker for Sjögren’s syndrome (SS). **(A)** Principal coordinate analysis of saliva samples from non-SS and SS subjects based on the Bray-Curtis distance method (*n* = 32). Eclipses represent 95% confidence interval. **(B)** Core microbiome analysis of saliva from non-SS subjects at the genus level. **(C)** Core microbiome analysis of saliva from SS subjects at the genus level. **(D)** DESeq2-driven differential features of the microbiome at the genus level. Positive values of log2 fold change indicate increased abundance in SS subjects. **(E)** Volcano plot showing differential ASVs derived from metagenomeSeq among SS and non-SS subjects. Gray dashed line indicates p-value cutoff (*p* = 0.05). Red and blue dashed lines indicate fold change cutoff (fold change = 2). **(F)** Intersection of data from DESeq2 and metagenomeSeq was illustrated in the Venn diagram.

Next, the ability of the abundance of microbial genera to distinguish between non-SS and SS subjects was examined. The principal coordinates analysis plot indicated that disease status contributed to 5.9% of PG saliva microbiota composition variation with statistical significance (**Figure 4A**). The microbiome in the non-SS group mainly comprised relatively diverse genera, such as *Brevundimonas*, *Streptococcus*, *Haemophilus*, and *Lactobacillus* (**Figure 4B**), whereas that in the SS group was characterized by increased abundance of *Streptococcus* and decreased microbial diversity (**Figure 4C**). To identify potential biomarkers to distinguish between SS and non-SS subjects, the following two different differential analysis methods were used: DESeq2 and metagenomeSeq. RNA sequencing-based DESeq2 analysis revealed that several genera exhibited differentially abundance between the non-SS and SS groups (**Figure 4D**). Furthermore, the metagenomeSeq-driven volcano plot revealed that the abundance of *Bacteroides vulgatus, Staphylococcus aureus*, *Methylorubrum extorquens*, *Blautia coccoides*, and *Lactobacillus johnsonnii* was significantly downregulated in the SS group (**Figure 4E**). Although a biomarker that was specifically enriched in the SS group was not identified, *Lactobacillus* was the common genus identified in DESeq2 and metagenomeSeq analyses. This suggested that the abundance of *Lactobacillus* is downregulated in SS subjects but not in non-SS subjects (**Figure 4F**).

**Figure 5:**
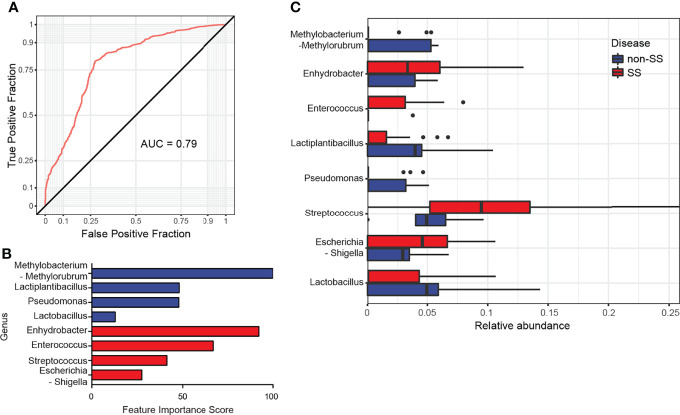
Microbiome features predicted with logistic regression as supervised learning classification algorithm. **(A)** Receiving operating characteristic curve of logistic regression using eight genera. The diagonal line represents an area under the curve value of 0.5, which was a random decision. **(B)** The feature importance scores of the top eight genera used in logistic regression were illustrated. Blue bars indicate non-Sjögren’s syndrome (SS) group-specific genera, while red bars indicate SS group-specific genera. **(C)** The relative abundance of the eight genera used in logistic regression was comparatively analyzed between non-SS and SS groups.

Furthermore, the logistic regression model was used to determine whether the abundance of the microbiome at the genus level could be used to predict SS. The model was constructed with the top eight genera with the area under the curve value of 0.79 (**Figure 5A**). Among the tested genera, four genera, including *Streptococcus*, exhibited featured importance scores in patients with SS. Similarly, four genera, including *Lactobacillus*, exhibited featured importance scores in non-SS subjects (**Figure 5B**). The relative abundance of each genus also exhibited a similar enrichment pattern based on the feature importance score (**Figure 5C**). This suggested that these genera can differentiate SS subjects from non-SS subjects.

### Dysbiosis Cluster Is Highly Enriched With *Streptococcus*

**Figure 6:**
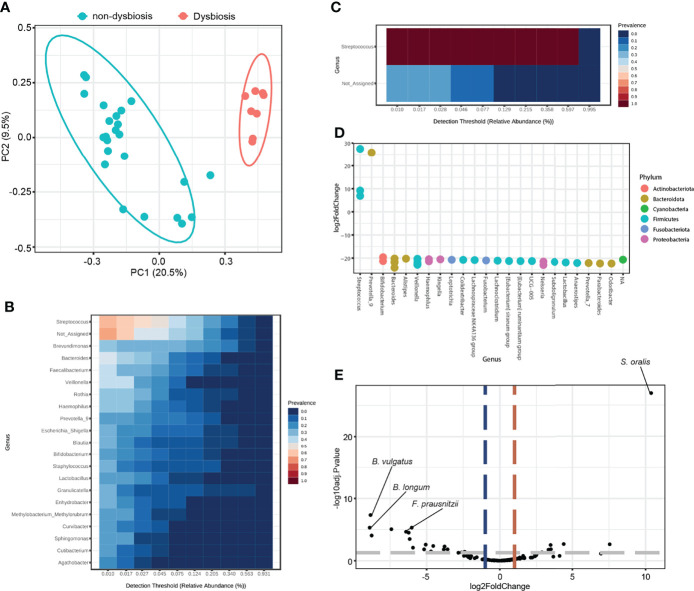
Differential analysis based on microbiome dysbiosis. **(A)** Principal coordinate analysis of saliva samples based on the Bray-Curtis distance method (*n* = 32). Red dots indicate samples in a distinct cluster, which was denoted as the dysbiosis group. Eclipses represent 95% confidence interval. **(B)** Core microbiome analysis of saliva from the non-dysbiosis group at the genus level. **(C)** Core microbiome analysis of saliva from the dysbiosis group at the genus level. **(D)** DESeq2-driven differential features of the microbiome at the genus level. Positive values of log2 fold change indicate increased abundance in the dysbiosis group. **(E)** Volcano plot showing differential ASVs derived from metagenomeSeq between the dysbiosis and non-dysbiosis groups. Gray dashed line indicates p-value cutoff (*p* = 0.05). Red and blue dashed lines indicate fold change cutoff (fold change = 2).

A large overlap in the microbial composition was observed between the non-SS and SS groups, while separate microbial clusters were observed in some SS subjects (**Figure 6A**). Thus, this cluster was termed the “dysbiosis cluster” and used for further analysis. The microbiome in the dysbiosis cluster was highly enriched with the genus *Streptococcus*, while various taxa were enriched in the non-dysbiosis cluster (**Figures 6B, C**). Differential analysis of DESeq2 and metagenomeSeq results revealed that the abundance of *Streptococcus oralis* was high, while the abundance of other species, including *Bacteroides vulgatus*, *Bifidobacterium longum*, and *Faecalibacterium prausnitzii*, was markedly downregulated in the dysbiosis cluster (**Figures 6D, E**). These findings indicate that *Streptococcus* and *Lactobacillus* are potential biomarkers for SS and non-SS subjects, respectively, at the genus level.

## Discussion

This study determined the correlation between the microbiome in the saliva, which was obtained through PG lavage, and clinical features of patients with SS sicca. The anti-SSA positivity, salivary flow, and sialectactic structural changes in laboratory and radiological examinations were correlated with microbiota composition and dysbiosis. SS subjects with sialectasis exhibited decreased microbial diversity and changes in the abundance of bacterial phyla, such as Firmicutes and Proteobacteria. SFR affected the abundance of Bacteroidetes, which subsequently affected microbiota diversity. DESeq2 and MetagonomeSeq analyses revealed that the SS sicca group exhibited distinct patterns of microbiome genera. The salivary abundance of *Lactobacillus* in the SS group was lower than that in the non-SS sicca group. Next, statistical and bioinformatics analyses were performed to determine whether the oral microbiome could be used as a potential predictor for SS sicca. The analysis revealed that *Lactobacillus* and *Streptococcus* are potential biomarkers for differentiating SS sicca and non-SS sicca subjects.

Dysbiosis is involved in the pathogenesis of autoimmune diseases by altering the oral, gut, and skin flora. The composition of the gut microbiota in patients with SS and systemic lupus erythematosus (SLE) is similar but distinct from that of population control (8, 30). Oral microbiome, which harbors more than 1,000 species (31), may play an important role in the onset or progression of SS. Recent studies have demonstrated that the oral microbiome in patients with SS is significantly different from that in healthy individuals (32, 33). Oral microbiota composition varies between patients with SS and patients with SLE (8). However, the composition of the oral microbiome varies depending on the location (34–36). Furthermore, microbial diversity in the oral cavity partly results from differences in the oral microenvironment, which is influenced by the diet and lifestyle of the host (11, 37). Local salivary gland dysfunction and systemic diseases also significantly affect the composition of oral microflora. Therefore, for better quality of data, enrollment of homogeneous cohorts and a standardized protocol for the sampling are required.

A recent meta-analysis assembled data regarding the oral and gut microbiota in SS and compared them between healthy individuals and patients with sicca symptoms without SS or SLE. The results provided evidence that sicca patients seem to be more relevant than healthy subjects as a control group to analyze differences in the microbiota (38). In our study, a non-SS sicca cohort was enrolled as a control group, and PG saliva was utilized to investigate microbial changes and establish the role of the microbiome in the pathogenesis of SS. PG lavage allows saliva to be collected directly from the salivary gland. Since saliva contains several antimicrobial compounds, including antimicrobial peptides, hydrogen peroxide, and lysozymes, we assume that the PG saliva contains less microbial content than whole mouth saliva. Although it is difficult to directly compare our data with previously reported population data, since DNA extraction, sequencing, and analysis were performed with different protocols, the microbiota composition of PG saliva may differ to that in oral microbiota from healthy or sicca patients. To confirm this, further studies employing 16S rRNA sequencing for whole saliva samples paired with PG saliva are warranted. Nevertheless, the saliva collection protocol and methodology utilized in this study make novel contributions to understanding salivary microbiome components.

The microbiome is composed of several phyla, including Firmicutes, Bacteroidetes, Actinobacteria, Proteobacteria, and Fusobacteria. Firmicutes and Bacteroidetes constitute 90% of the gut microbiome (39). Consistently, the top four phyla in the PG saliva microbiome were Firmicutes (49.69%), Proteobacteria (23.71%), Bacteroidetes (14.01%), and Actinobacteria (8.18%), which constituted approximately 95% of the PG microbiome. Thus, the correlation between the clinical features of SS and the abundance of the top four phyla was examined in this study. Microbial diversity was significantly correlated with anti-SSA positivity and PG ductal type. The differential composition of phyla between the SS sicca and non-SS sicca groups suggested that a specific phylum can be used to predict and monitor SS. The phyla Firmicutes, Proteobacteria, and Actinobacteria were significantly correlated with anti-SSA/Ro positivity. These findings suggest that the microbiome composition may indicate the biological status in SS subjects. The microbiome composition can be a predictive marker for SS considering the diagnostic variability of autoantibodies. However, microbial profiles were not significantly correlated with positive LSGB results. The sample numbers used in this study may not provide significant results. Additionally, LSGB possess inherent sampling-related issues. For example, when the patients lack minor salivary glands in the lips, possibly due to severe atrophy, the number of glands may be insufficient to evaluate immune reaction. Further studies are needed to determine the correlation between microbial features and autoimmune profiles.

MR sialography revealed that sialectasis in PG ducts and fat deposition in SMGs were the characteristic features of the SS sicca group. Apple tree-like appearance resulting from sialectactic structural changes was often observed in the PG ducts (40). Consistently, the microbiome composition was significantly correlated with the radiological findings of the SS sicca group. The sialectasis-like dilated duct may be induced by an immune reaction in the salivary glands. In this case, the microbiome may contribute to the development of structural deformity by eliciting secondary inflammatory responses of the salivary ductal epithelial cells against salivary microbiome. Microbial enrichment or dysbiosis can also occur because of ductal dilation and consequent saliva stasis in the sialectactic lesion. To address whether microbiota have a protective or provocative role in autoimmunity, the host-microbiota interactions must be elucidated. However, the significant correlation of structural changes in PGs with the composition of Firmicutes and F/B ratio in PG saliva suggests that the microbiome has a potential role in the pathogenesis of SS. Recent clinical studies have reported that fatty degeneration of salivary glands might help to diagnose SS, as well as evaluate functional disease status (41). In this study, the fat deposition stage in SMGs on MR sialography was positively correlated with the Shannon index, suggesting that fatty degeneration may reflect structural deformity-related disease status and microbial diversity in SS.

The salivary gland is infected in pathological conditions, including sialadenitis, salivary stones, or other duct blockages (42). The facultatively anaerobic environment of the oral cavity in pathological condition (43) promotes salivary stone-mediated formation of biofilms in which *Streptococcus oralis* is enriched (44). The findings of this study indicate that *S. oralis* is a major dysbiotic microbe in the salivary glands (**Figure 6E**). The enrichment of *S. oralis* may result from duct blockage. However, *S. oralis* can also directly contribute to the progression of SS by promoting H_2_O_2_-mediated killing of human macrophages and epithelial cells (45). Previous studies have reported the protective and homeostatic roles of *Lactobacillus* (46, 47). We hypothesized that *Lactobacillus* regulates the microbiome and immune balance in the salivary gland through the expansion of regulatory T cells (48) or the production of anti-microbial peptides (49). Adoptive transfer of specific microbes into experimental SS mice (50) will enable the identification of the direct role of the microbiome in SS pathophysiology.

This study is associated with several limitations. PG saliva may contain some microbial content, which can affect the interpretation of the results and yield low sequencing reads. Additionally, the results may be skewed as the abundant microbes are easily detected, whereas the rare microbial populations may be missed (51, 52). Recently, low-abundance microorganisms were reported to impact the dysbiotic signatures of local microbial habitats (53). Thus, the possibility of rare microbial species regulating the microbiome in the salivary gland cannot be ruled out. Furthermore, the number of enrolled subjects was low, particularly for non-SS sicca in the study cohort owing to the long-lasting COVID-19 pandemic, which was a major hurdle for collecting saliva from patients and performing other clinical trials (54). Likewise, the number of patients who underwent measurement of salivary flow rate in the non-SS group was low (n = 3), and one individual showed relatively higher SFR than others. Xerostomia is a subjective terminology and is not the same as salivary gland hypofunction. We suspect that one patient who showed high SFR might have had borderline salivary gland dysfunction. We suspect that further studies with a large sample size and high-throughput sequencing with increased sequencing depth will aid in identifying microbial dysregulation in the salivary glands of patients with SS.

In conclusion, the findings of this study indicate that the salivary gland microbiome is involved in the pathogenesis and clinical progression of SS. Future studies must focus on screening microbial biomarkers for SS. The findings of this study will enable the application of precision medicine and the development of management strategies, such as probiotics and dietary interventions to target the microbiome for patients with SS.

## Data Availability Statement

The original contributions presented in the study are publicly available. This data can be found here: https://www.ncbi.nlm.nih.gov/search/all/?term=PRJNA804331

## Ethics Statement

The studies involving human participants were reviewed and approved by The Institutional Review Board of the Gangnam Severance Hospital. The patients/participants provided their written informed consent to participate in this study.

## Author Contributions

DK: Contributed to conceptualization, investigation, formal analysis, and visualization, drafted and revised the manuscript. YJJ: Contributed to investigation, formal analysis, and data curation. YL: Contributed to investigation and data curation. JC: Contributed to formal analysis and resources. YMP: Contributed to investigation. OCK: Contributed to resources. YWJ : Contributed to resources. SJA: Contributed to formal analysis and revised the manuscript. HKL: Contributed to resources. M-CP: Contributed to resources. J-YL: Contributed to conceptualization, supervision, funding acquisition, and drafted and revised the manuscript. All authors gave their final approval and agreed to be accountable for all aspects of the work.

## Funding

This research was supported by the Bio & Medical Technology Development Program of the National Research Foundation (NRF) funded by the Ministry of Science & ICT (NRF-2020M3A9I4039045) and the Research Grant from Gangnam Severance Hospital, Yonsei University College of Medicine.

## Conflict of Interest

The authors declare that the research was conducted in the absence of any commercial or financial relationships that could be construed as a potential conflict of interest.

## Publisher’s Note

All claims expressed in this article are solely those of the authors and do not necessarily represent those of their affiliated organizations, or those of the publisher, the editors and the reviewers. Any product that may be evaluated in this article, or claim that may be made by its manufacturer, is not guaranteed or endorsed by the publisher.
