# Supplementary material for: Correlation Between Salivary Microbiome of Parotid Glands and Clinical Features in Primary Sjögren’s Syndrome and Non-Sjögren’s Sicca Subjects
Source: Front Immunol. 2022 May 4;13:874285. doi: 10.3389/fimmu.2022.874285 (PMC9114876; doi:10.3389/fimmu.2022.874285)
Supplement: Supplementary file 1 [file DataSheet_1.pdf]

Supplementary Material

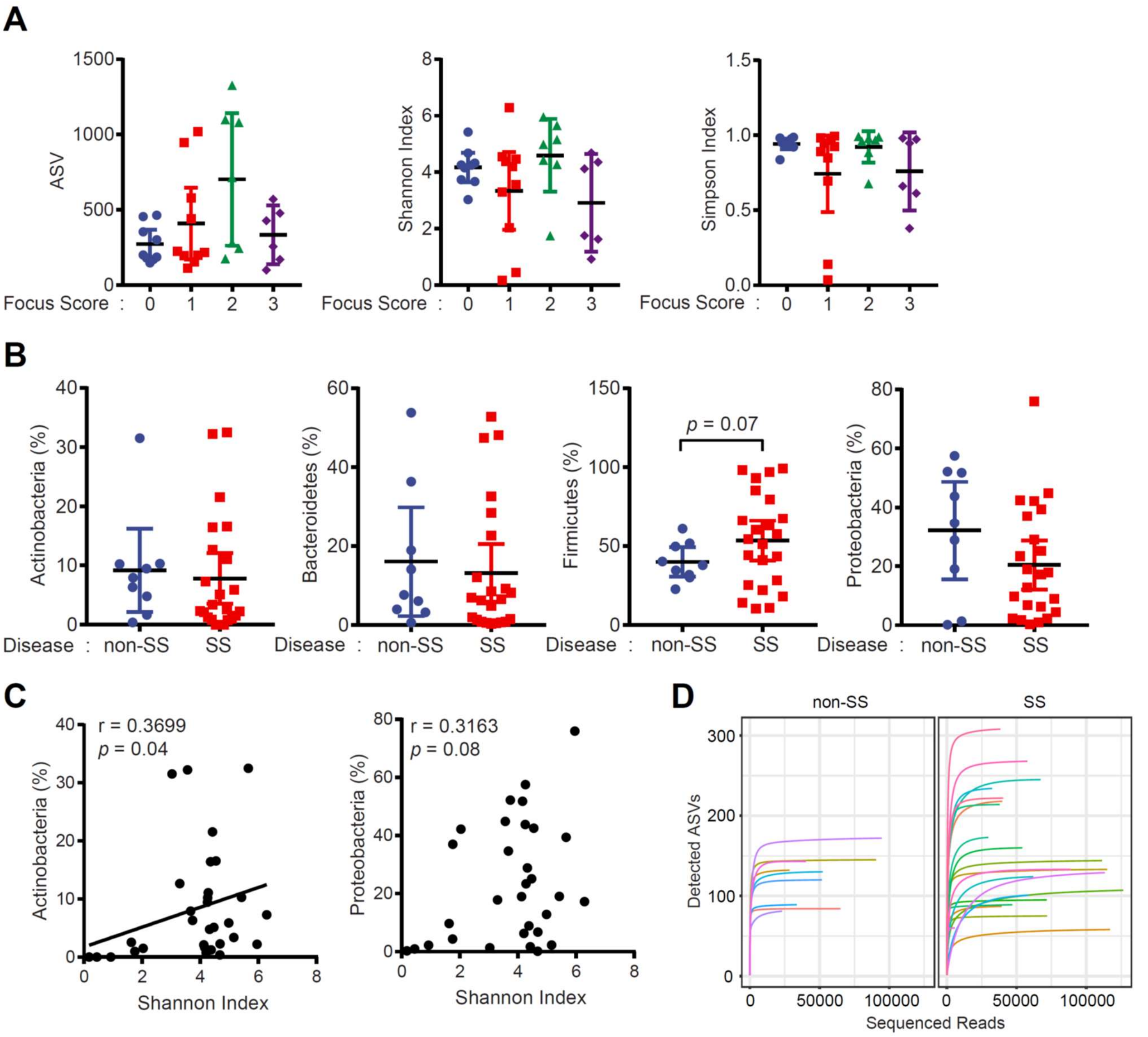

Supplementary Figure 1. Related to Figure 2. The alpha-diversity and the abundance of each phylum in patients with Sjögren's syndrome (SS) and non-SS sicca. (A) Amplicon sequence variant (ASV) (left), Shannon index (center), and Simpson index (right) in the saliva samples based on the focus scores 0 (n = 9), 1 (n = 10), 2 (n = 7), and 3 (n = 6). **(B)** The abundances of the top four phyla in the non-SS (n = 9) and SS groups (n = 23). **(C)** Correlation between the Shannon index and the abundance of Actinobacteria (left) or Proteobacteria (right) (n = 32). **(D)** Rarefaction curves for non-SS (left) and SS (right) samples. Data are represented as mean ± 95% confidence interval.

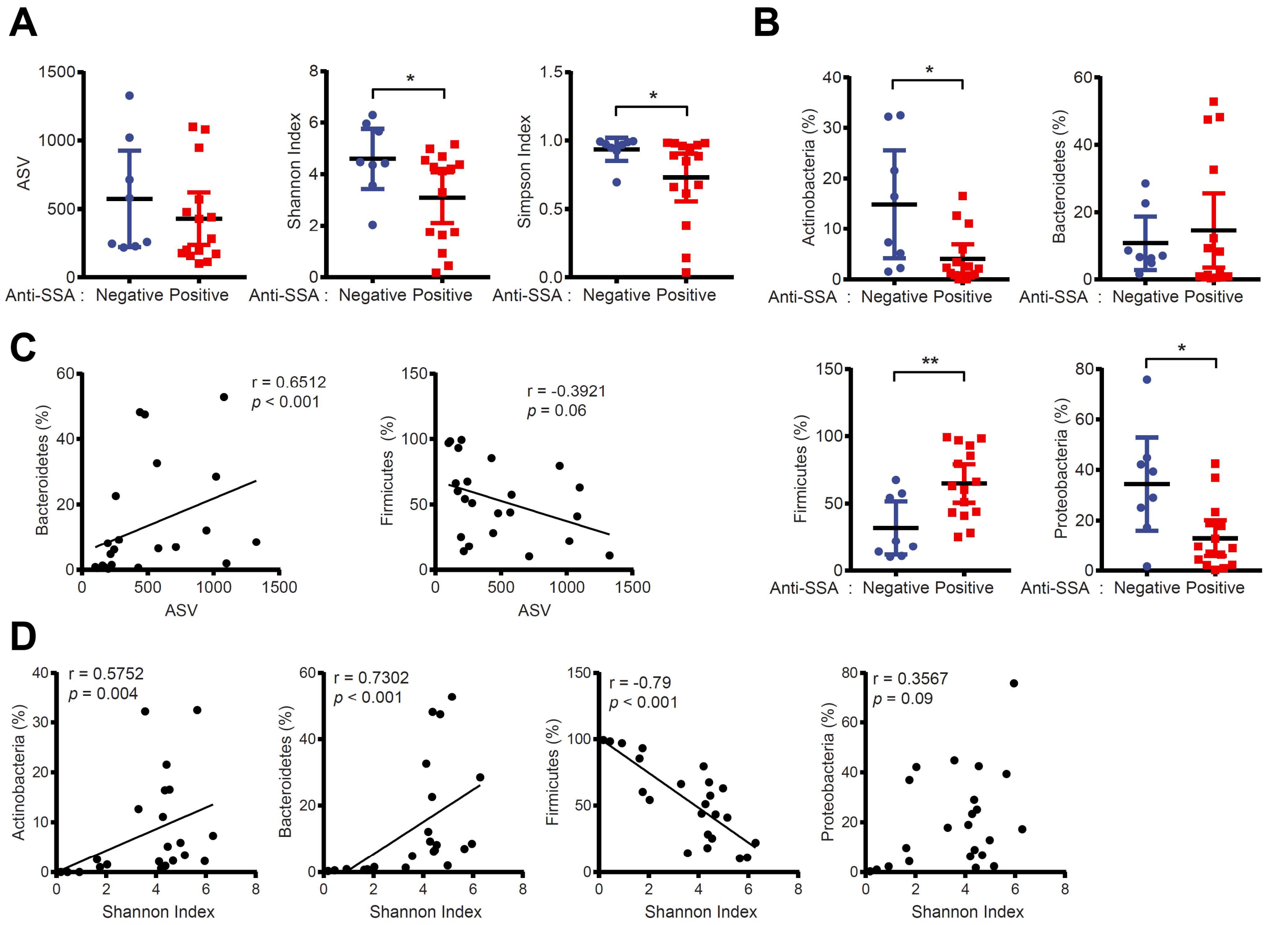

Supplementary Figure 2. Related to Figure 2. The alpha-diversity and the abundance of each phylum in SS group only. (A) ASV (left), Shannon index (center), and Simpson index (right) in the saliva samples based on anti-SSA-negative ($n = 8$) and -positive ($n = 15$) cohorts from SS group. **(B)** The abundances of the top four phyla in the anti-SSA-negative and -positive cohorts from SS group. **(C)** Correlation between the ASV and the abundance of Bacteroidetes (left) or Firmicutes (right) in SS group ($n = 23$). **(D)** Correlation between the Shannon index and the abundance of top four phyla in SS group ($n = 23$). Data are represented as mean ± 95% confidence interval. * $p < 0.05$, ** $p < 0.01$.

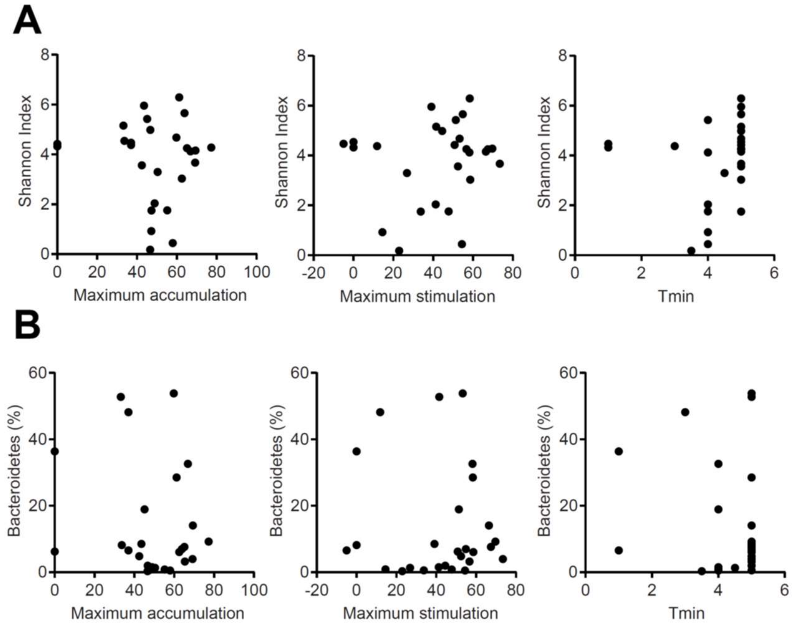

Supplementary Figure 3. Related to Figure 3. Correlation between scintigraphy parameters and microbial composition. (A) Correlation analysis between scintigraphy parameters (maximum accumulation, left; maximum stimulation, middle; Tmin; right) and Shannon index (*n* = 27). **(B)** Correlation analysis between scintigraphy parameters (maximum accumulation, left; maximum stimulation, middle; Tmin; right) and abundance of Bacteroidetes phylum (*n* = 27).
